# Modulation effects of repeated transcranial direct current stimulation on the dorsal attention and frontal parietal networks and its association with placebo and nocebo effects

**DOI:** 10.1016/j.neuroimage.2023.120433

**Published:** 2023-11-06

**Authors:** Valeria Sacca, Ya Wen, Sierra Hodges, Jian Kong

**Affiliations:** Department of Psychiatry, Massachusetts General Hospital and Harvard Medical School, Charlestown, MA 02129, USA

**Keywords:** Dorsal attention network, Dorsolateral prefrontal cortex, Frontoparietal network, Nocebo hyperalgesia, Placebo analgesia, Transcranial direct current stimulation

## Abstract

Literature suggests that attention is a critical cognitive process for pain perception and modulation and may play an important role in placebo and nocebo effects. Here, we investigated how repeated transcranial direct current stimulation (tDCS) applied at the dorsolateral prefrontal cortex (DLPFC) for three consecutive days can modulate the brain functional connectivity (FC) of two networks involved in cognitive control: the frontoparietal network (FPN) and dorsal attention network (DAN), and its association with placebo and nocebo effects.

81 healthy subjects were randomized to three groups: anodal, cathodal, and sham tDCS. Resting state fMRI scans were acquired pre- and post- tDCS on the first and third day of tDCS. An Independent Component Analysis (ICA) was performed to identify the FPN and DAN. ANCOVA was applied for group analysis.

Compared to sham tDCS, 1) both cathodal and anodal tDCS increased the FC between the DAN and right parietal operculum; cathodal tDCS also increased the FC between the DAN and right postcentral gyrus; 2) anodal tDCS led to an increased FC between the FPN and right parietal operculum, while cathodal tDCS was associated with increased FC between the FPN and left superior parietal lobule/precuneus; 3) the FC increase between the DAN and right parietal operculum was significantly correlated to the placebo analgesia effect in the cathodal group.

Our findings suggest that both repeated cathodal and anodal tDCS could modulate the FC of two important cognitive brain networks (DAN and FPN), which may modulate placebo / nocebo effects.

## 1. Introduction

In the last few years, brain imaging has been widely used to explore the umderlying mechanisms of placebo and nocebo effects. However, most of the current studies have applied an observational method, investigating the brain networks associated with placebo and nocebo effects. Few studies have explored how the excitability/functional connectivity (FC) changes of certain brain regions could modulate the placebo and nocebo effects.

Placebo analgesia and nocebo hyperalgesia are the most well studied placebo/nocebo effects. Literature suggests that pain experience involve multiple brain networks functioning in the sensory, affective, and cognitive aspects of pain signal processing (Garland, 2012; Wiech, 2016). Furthermore, studies have also shown that attention is a critical cognitive process for pain perception and modulation (Bantick et al., 2002) and plays an important role in placebo and nocebo effects (Gibbons et al., 1979).

Multiple brain networks are involved in cognitive control. One of them is the frontoparietal network (FPN) which is involved in perceptual attention, information manipulation in working memory, and goal-directed tasks (Marek and Dosenbach, 2018). Another network is the dorsal attention network (DAN) which is engaged in top-down controlled attentional selection for stimuli and responses (Corbetta and Shulman, 2002; Vossel et al., 2014). The FPN connects to the DAN functioning in the regulation of perceptual attention (Dixon et al., 2018). Increased placebo activities have been found in the FPN, as well as in the anterior intraparietal sulcus which is part of the DAN (Zunhammer et al., 2021).

Furthermore, literature suggests that the dorsolateral prefrontal cortex (DLPFC), a key region in the FPN, plays a crucial role in pain processing and modulation (Lorenz et al., 2003; Seminowicz and Moayedi, 2017). Recently, studies have shown that the DLPFC may modulate placebo analgesia and nocebo hyperalgesia through the transcranial magnetic stimulation (TMS) and transcranial direct current stimulation (tDCS) as expectancy manipulation models (Egorova et al., 2015; Krummenacher et al., 2010; Tu et al., 2021b). TMS and tDCS are both types of noninvasive brain stimulation that can modulate the cortical excitability and induce prolonged after-effects in the brain that could influence behavior (Fregni et al., 2021; Kluger and Triggs, 2007). Unlike TMS, tDCS does not stimulate nerve cells or activate regions of the brain (Nitsche and Paulus, 2000; Siebner et al., 2009). In a previous study (Tu et al., 2021b), we investigated how tDCS at the right DLPFC (rDLPFC) can modulate placebo and nocebo effects and the brain response to calibrated experimental heat pain. An expectancy manipulation model was used to induce positive and negative expectations in healthy participants using three inert creams: (i) lidocaine for inducing expectations of decreased pain; (ii) capsaicin for the expectations of increased pain; and (iii) neutral moisturizer as a control. The healthy participants were randomized into three tDCS groups (anodal, cathodal, and sham), and tDCS was applied for three consecutive days. For the assessment of placebo and nocebo effects, heat pain stimuli were performed with the application of the three inert creams on the subjects’ forearm. Placebo and nocebo effects were assessed by calculating the pain rating difference between identical moderate painful stimuli applied on areas of skin with lidocaine cream and those applied on areas of skin with neutral cream. Nocebo hyperalgesia was defined as pain rating differences between identical moderate painful stimuli applied on areas of skin with capsaicin cream and those applied on areas of skin with neutral cream. We found that in comparison with the sham group, cathodal tDCS increased placebo analgesia while the anodal tDCS inhibited nocebo hyperalgesia. Moreover, we found that cathodal and anodal tDCS may modulate brain activity and connectivity which are correlated with the placebo and nocebo effects (such as the insula and ventromedial prefrontal cortex (vmPFC)). These results suggest that changing the excitability of the DLPFC through tDCS may modulate placebo and nocebo effects, which could have important implications in the clinical field.

With this in mind, we pursued a follow up study investigating how different tDCS treatments (cathodal, anodal, and sham) for three consecutive days can modulate the resting state FC of the FPN and DAN, and the associations between these changes and placebo/nocebo effects. We hypothesize that tDCS at the rDLPFC can modulate the FC of the frontoparietal and dorsal attention networks, and that these functional changes are associated with placebo and nocebo effects. Elucidating the modulation effects of tDCS may help to understand the mechanisms of placebo/nocebo effects and develop new strategies to harness these effects.

## 2. Materials and methods

### 2.1. Participants

We recruited 103 healthy participants at Massachusetts General Hospital (MGH) from September 2016 to March 2019. Twenty-two participants were dropped from the study: 18 before randomization and 4 after randomization due to scheduling issues or a device error (3 from the cathodal group, and 1 from the sham). The final number of participants was 81 (37 females, mean ± SD age: 27.4 ± 6.4), with 27 participants in each of the three tDCS groups.

The study was approved by the MGH Institutional Review Board. Informed consent was obtained from all participants. Please see our previous publication for more details (Sacca et al., 2023; Tu et al., 2021b, 2021a).

### 2.2. Experimental procedure

Each participant attended five sessions. Session 1 was a training, familiarity, and calibration session. Subjects were trained to use the Sensory Gracely Scale (0–20) (Gracely et al., 1978a, 1978b) to rate pain experiences using the same method as in our previous studies (Freeman et al., 2015; Kong et al., 2008, 2006). Subjects first received an ascending heat stimulus sequence (starting from 38 °). The three temperatures that each subject rates as approximately 5–6 (mild pain), 10–11 (moderate pain) and 14–15 (strong pain) were selected.

Session 2 was an expectancy manipulation session. At the beginning of the session, all subjects were informed that the aim of this study was to use a neuromodulation tool (tDCS) to investigate the analgesic effects of lidocaine cream and the hyperalgesic effects of capsaicin cream using a neutral cream as a control. We first applied sham tDCS for 20 min, and then applied all three creams to different spots on participants’ right volar forearm. Subjects were told that the creams needed about 20 min to take effect and that the effect will last for more than two hours. In reality, only sham tDCS was applied, and an inert cream was used for all three creams. The cream was a fragrance-free moisturizing lotion dyed three different colors (blue for “Lidocaine”, pink for “Capsaicin”, and white for “Neutral”).

**Fig. 1.**
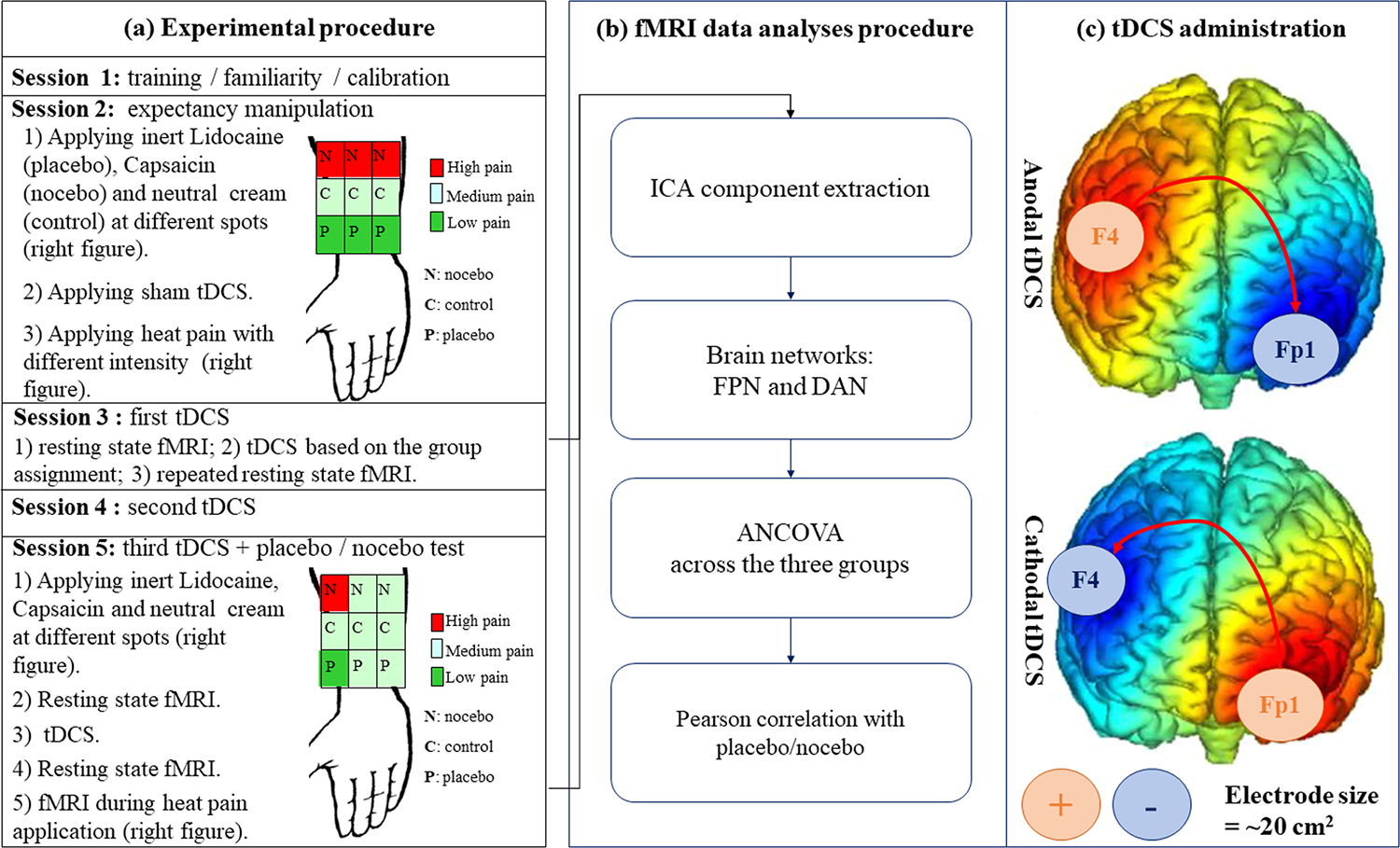
(a) experimental procedure and design of the study; (b) fMRI data analyses procedure; (c) anodal and cathodal tDCS configurations.

After this scripted explanation, 9 unique regions of the subject’s arm were demarcated for each stimulus. We have drawn a 3 × 3 grid comprised of about 2 cm × 2 cm squares on the subject’s right volar forearm, starting the grid at the subject’s elbow crease. The creams were then applied with each cream spread onto a unique set of 3 adjacent squares (i.e., one cream for each row, Fig. 1(a)).

Using methods similar to a previous study (Freeman et al., 2015), subjects were then told that to test the hyperalgesia effect of capsaicin and the analgesia effect of the lidocaine, 6 identical pain stimuli would be applied to each of the nine spots. However, in reality, to boost subjects’ expectancy, 6 moderate stimuli were given to each of the neutral control cream spots, 6 mild stimuli were applied on placebo Lidocaine cream spots, and 6 high stimuli were applied on placebo Capsaicin cream spots (Fig. 1(a)). The location of the placebo, nocebo and control rows were randomized across subjects to balance the experimental design.

Session 3 was the first tDCS session. Right before and after tDCS, resting state fMRI data were collected. Session 4 was the second tDCS session with no fMRI scan involved. Session 5 was the third tDCS and placebo/nocebo test session. At the beginning of this session, subjects were told that the Session 2 procedure would be repeated in the MRI scanner. As in Session 2, the three different creams (in reality all one inert cream) were administered to each row of squares, with “Lidocaine” and “Capsaicin” cream administered to the same rows as determined in Session 2. Then, tDCS was applied based on subjects’ randomization. The resting state fMRI data were collected before and after tDCS.

After that, calibrated pain was applied during the fMRI scan. Since this session aimed to test the placebo/nocebo effect, we only administered different heat stimuli to the 3 regions in one column of the 9 × 9 grid. Then, subjects were asked to rate their expectancy of both the analgesia effect of Lidocaine cream and the hyperalgesic effect of Capsaicin using a 0–10 scale. After that, we administered the same moderate stimulus to all remaining regions with all 3 creams (Fig. 1(a)). Our outcome measurement was the subjective pain ratings to identical calibrated heat pain.

At the end of the experiment, subjects were debriefed. We informed them that in this study, only inert cream and sham tDCS (in session 2 or in sham tDCS group) was used, and that we changed the heat pain temperature at session 2. We also emphasized that the experience of this study should not influence their impression of Lidocaine/Capsaicin or their interest in choosing these drugs as a potential therapeutic method.

### 2.3. tDCS administration

The tDCS was applied on three consecutive days (the first and last sessions were performed inside the MRI scanner, collecting fMRI data before, during, and after the stimulation), and participants were randomized into one of three groups: cathodal tDCS, anodal tDCS, and sham tDCS. Anodal and cathodal configurations are reported in Fig. 1(c). The tDCS modes were configured inside the device system and were blinded for the operators/analysts and participants.

For each tDCS session, tDCS was applied at 2 mA for 20 min using the StarStim system (Neuroelectrics, Spain) through MRI-compatible electrodes (contact surface of about 20 cm^2^) for stimulation of the rDLPFC and the left orbitofrontal cortex (lOFC). In the anodal group, the anodal electrode was placed over F4 and the cathodal one over Fp1 (based on 10–20 EEG system) to inhibit the lOFC excitability and enhance the rDLPFC excitability. In the cathodal group, the cathodal electrode was located over F4 and the anodal one over Fp1 to inhibit the rDLPFC and enhance the lOFC excitability. For sham tDCS, the electrodes were placed at the same positions, and the current was applied just for 15 s both at the beginning and at the end of a 20 min sham-stimulation period to simulate the stimulation through a local tingling sensation. The impedances were kept below 10 kΩ for both stimulation electrodes.

### 2.4. MRI acquisition

MRI data were acquired at MGH Martinos Center for Biomedical Imaging using a 32-channel radiofrequency head coil in a 3T Siemens scanner (Siemens 3T Skyra). Structural brain images were acquired using a T1-weighted three-dimensional multiecho magnetization-prepared rapid gradient-echo sequence (voxel size: 1 × 1 × 1 mm3, repetition time: 2500 ms, echo time: 1.69 ms, slice thickness 1 mm, flip angle: 7 °, and 176 slices; measurement time: ~6 min).

On day 1 and day 3, four resting-state fMRI (rs-fMRI) sequences were acquired: (i) one before the tDCS application; (ii) two during the application of the tDCS; (iii) one shortly after the tDCS application. A whole-brain gradient-echo-planar-imaging sequence was used for functional scanning (voxel size: 3 × 3 × 3 mm^3^, repetition time: 3000 ms, echo time: 30 ms, slice thickness: 2.6 mm, flip angle: 90 °, and 44 slices), with a total of 125 vol collected. The MRI protocol during the tDCS application included: two rs-fMRI (~ 6 min for each scan) and one pulsed continuous arterial spin labeling (pCASL) sequence (~ 6 min).

To apply the tDCS during the fMRI scans, we used the Neuroelectrics Multi-Channel MRI Extension Kit, which allows us to connect the device (outside the MRI room) to the participants in the MRI scanner, safely obtaining high-quality scanning during stimulation.

### 2.5. MRI preprocessing

MRI data quality was checked using mriqc (Esteban et al., 2017) and Temporal Signal-to-Noise Ratio (tSNR) of the rs-fMRI sequences (see Supplementary Materials and Figure S1).

MRI data were pre-processed using fMRIprep version 20.0.4 (Esteban et al., 2019). T1-weighted images were corrected for intensity non-uniformity (INU) with N4BiasFieldCorrection (Tustison et al., 2010) and skull-stripped with the antsBrainExtraction.sh. Brain tissue segmentation of cerebrospinal fluid (CSF), white-matter (WM) and gray-matter (GM) was performed on the brain-extracted T1w using FSL (version 5.0.9) toolbox, FAST (Zhang et al., 2001).

Brain surfaces were reconstructed using recon-all (FreeSurfer 6.0.1; Fischl et al., 1999), and the brain mask estimated previously was refined with a custom variation of the method to reconcile ANTs-derived and FreeSurfer-derived segmentations of the cortical gray-matter of Mindboggle (Klein et al., 2017). Volume-based spatial normalization to one standard space (MNI152NLin2009cAsym) was performed through nonlinear registration with antsRegistration (ANTs 2.2.0), using brain-extracted versions of both T1w reference and the T1w template.

The rs-fMRI scans were preprocessed using the following default pipeline. First, a reference volume and its skull-stripped version were generated. The BOLD reference was then co-registered to the T1w reference using bbregister (FreeSurfer) which implements boundary-based registration (Greve and Fischl, 2009). Head-motion parameters were estimated using MCFLIRT (FSL version 5.0.9, Jenkinson et al., 2002), and BOLD runs were slice-time corrected (Cox and Hyde, 1997) FNI 20,160,207 (Cox and Hyde, 1997). The BOLD time-series were resampled onto their original, native space by applying the transforms to correct for head-motion, and then, the time-series were resampled into standard space.

Several confounding time-series were calculated based on the preprocessed BOLD: framewise displacement (FD), derivative of RMS variance over voxels (DVARS) and three region-wise global signals. FD and DVARS are calculated for each functional run, both using their implementations in Nipype (Power et al., 2014). The three global signals are extracted within the CSF, the WM, and the whole-brain masks. Additionally, a set of physiological regressors were extracted to allow for component-based noise correction (CompCor; Behzadi et al., 2007). Principal components are estimated after high-pass filtering the preprocessed BOLD time-series (using a discrete cosine filter with 128 s cut-off) for the two CompCor variants: temporal (tCompCor) and anatomical (aCompCor). tCompCor components are then calculated from the top 5 % variable voxels within a mask covering the subcortical regions. This subcortical mask is obtained by heavily eroding the brain mask, which ensures it does not include cortical GM regions. For aCompCor, components are calculated within the intersection of the mask and the union of CSF and WM masks calculated in T1w space, after their projection to the native space of each functional run (using the inverse BOLD-to-T1w transformation). Components are also calculated separately within the WM and CSF masks.

The confound time series derived from head motion estimates and global signals were expanded with the inclusion of temporal derivatives and quadratic terms for each (Satterthwaite et al., 2013). Frames that exceeded a threshold of 0.5 mm FD or 1.5 standardized DVARS were annotated as motion outliers.

### 2.6. Independent components analysis and brain networks

Independent Component Analysis (ICA) was performed using CONN (version 21a; Calhoun et al., 2001). Similar to previous studies (Roberts et al., 2022; Saccà et al., 2023, 2019; Tu et al., 2019), entire sample (all data collected across the three groups, including all time-points) was used to compute one ICA to obtain the ICA maps for the following analysis. The group-ICA was performed using G1 FastICA for the estimation of the independent spatial components and GICA 3 Back-projection for the estimation of the individual subject-level spatial map.

Since this study’s aim was exploratory and the literature showed that model orders ≤ 20 provide the general picture of the brain networks (Abou-Elseoud et al., 2010; Elseoud et al., 2011), the number of the components was set at 20, and the dimensionality reduction factor was set at 64.

The ICA maps obtained were correlated with the ICA maps inside CONN toolbox (Calhoun et al., 2001) in order to identify the components of the networks. As in previous studies (Kornelsen et al., 2020; Prawiroharjo et al., 2020; Soman et al., 2020), after FPN and DAN networks were identified, differences between the three groups among the two networks were assessed using ANCOVA to compare the pre tDCS on day 1 (baseline) and post tDCS on day 3 (after the 3rd tDCS treatment) rs-fMRI changes across the whole brain for evaluating the cumulative effects of repeated tDCS. The comparisons between the groups allow us to estimate differences located within the same network (within-network connectivity) and/or outside this network (between--network connectivity, i.e., the connectivity between this network and the rest of the brain). Also, since placebo and nocebo tests were performed after the tDCS on day 3, the analysis will also allow us to explore the association between the connectivity changes and the placebo/nocebo effects.

For the whole brain analysis, a threshold of voxel-wise *p* < 0.005 and pFDR ≤ 0.05 at cluster level was applied. Age and gender were used as covariates.

### 2.7. Correlations

To assess the clinical importance of the ICA findings, Pearson correlation was performed between the average z-score values (extracted at the peak MNI) of the significant clusters and placebo analgesia and nocebo hyperalgesia. The placebo analgesia effect was calculated as the difference between perceived intensity to identical moderate painful stimuli applied on areas of skin with lidocaine cream and those applied on areas of skin with neutral cream. The nocebo hyperalgesia effect was extracted as the difference between the perceived intensity to identical moderate painful stimuli applied on areas of skin with capsaicin cream and on areas of skin with neutral cream.

Furthermore, we also extracted the average z-score values of the significant clusters using just the post tDCS on day 3 (the end of the treatment). Then, we performed another Pearson correlation between those clusters and placebo analgesia. Correlation analysis was performed using R software (Team, 2000). Bonferroni correction was performed for the multiple comparison for both correlation analyses.

Analyses that were conducted in this study are reported in Fig. 1(b).

## 3. Results

### 3.1. Participants

All 81 participants who completed the study were included in the analysis. Demographic parameters and behavioral results are reported in the Supplement Materials and Table S1 and S2. For more details, please refer to our previous publication (Tu et al., 2021b). In summary, the comparison between the three groups had significant main effects of the active tDCS groups on placebo analgesia (i.e., lidocaine versus neutral; *p* = 0.034) and nocebo hyperalgesia (i.e., capsaicin versus neutral; *p* = 0.044). Post-hoc comparisons revealed that cathodal tDCS significantly increased placebo analgesia in comparison to the sham group (p_Tukey_ = 0.028), and that anodal tDCS significantly inhibited nocebo hyperalgesia compared to the sham group (p_Tukey_ = 0.041).

### 3.2. Independent components analysis and brain networks

**Fig. 2.**
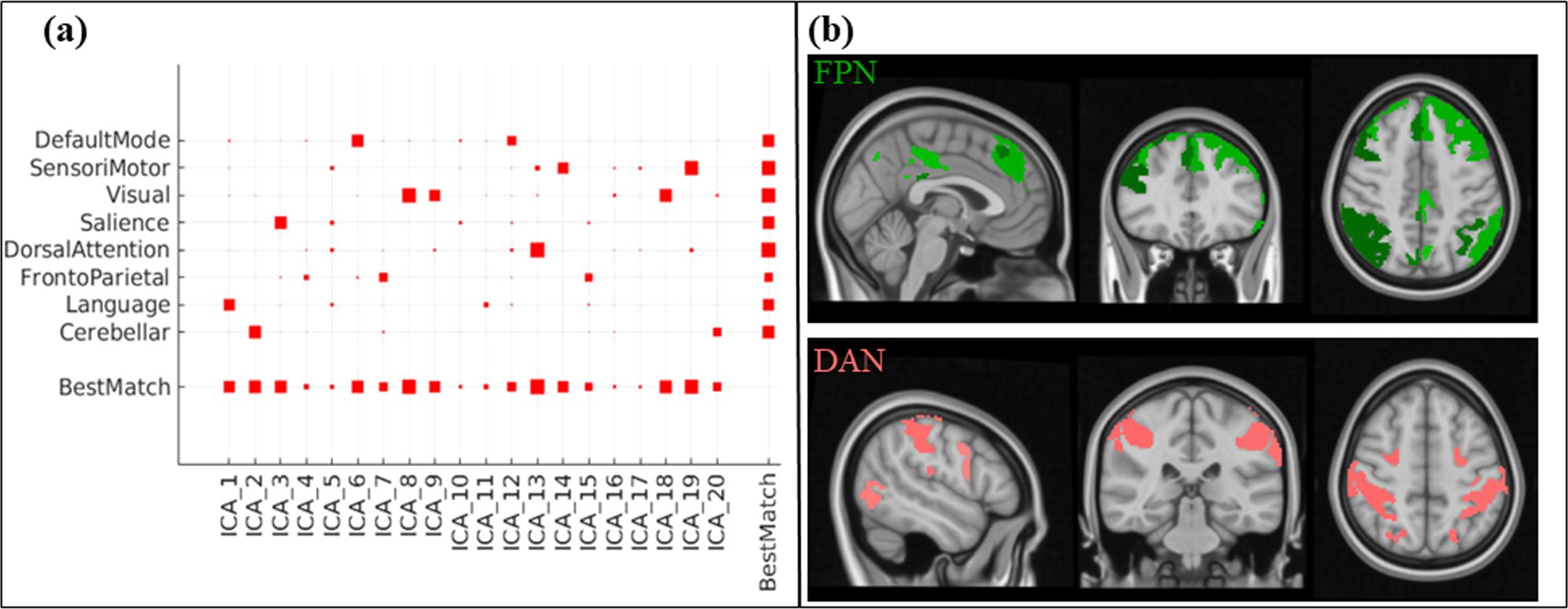
(a) Correlation between our ICA maps and the ICA maps inside of CONN; (b) The brain regions of the FPN (green) and DAN (pink).

Based on the ICA analysis, we identified the FPN and DAN by evaluating the correlation between our set of ICA and the ICA maps inside CONN (see Fig. 2(a)). In particular, the DAN was composed of just one component, while the FPN was composed of three different components. The brain regions of the FPN and DAN are shown in Fig. 2(b).

### 3.3. Dorsal attention network (DAN) results

Both active tDCS modes (anodal and cathodal) in comparison to the sham tDCS showed increased FC between the DAN and right operculum (parietal lobule) after repeated tDCS. The cathodal group also showed a significant FC increase between the right postcentral gyrus and the DAN.

**Fig. 3.**
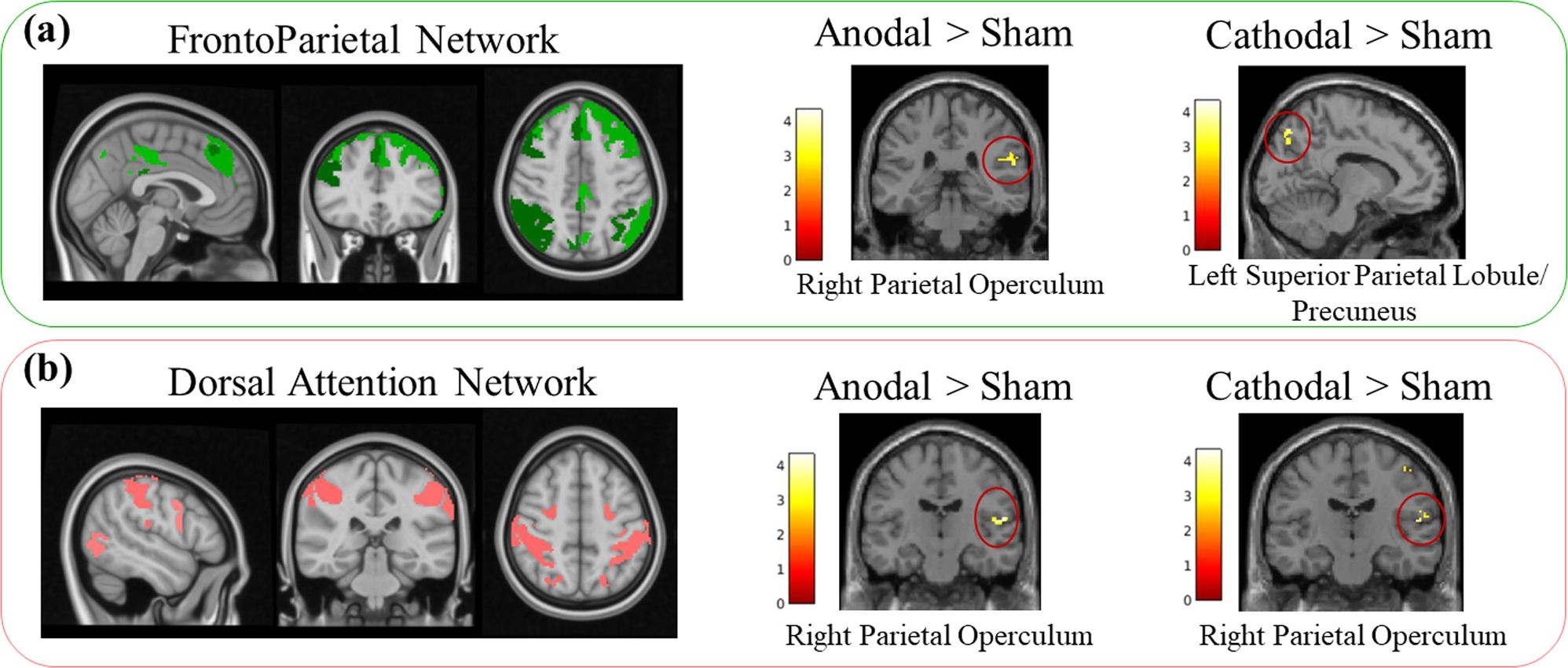
Identified Frontoparietal network (a) and Dorsal Attention network (b) and the clusters that showed significant differences from between-group comparison results.

No other significant findings were found (Fig. 3(b) and Table 1).

### 3.4. Fronto parietal network (FPN) results

The anodal group showed an increase in the FC between the FPN and right parietal operculum after tDCS in comparison to the sham tDCS, while the cathodal mode was associated with increased FC between the FPN and left superior parietal lobule/precuneus compared to the sham tDCS.

No other significant findings were found. Results are shown in Fig. 3 (a) and Table 1.

### 3.5. Correlations

We performed Pearson correlation analyses between placebo analgesia and the average z values of the significant clusters found in the ICA analysis. We found a significant positive correlation between the right parietal operculum and DAN (the cluster showed a significant difference between the cathodal and sham comparisons) FC change (pre minus post tDCS) and the corresponding placebo analgesia (*r* = 0.49, *p* < 0.001; significant after Bonferroni correction (*p* = 0.008 (0.05/6)) across all participants. No other significant results were found.

Furthermore, we performed the correlation between the DAN-operculum FC change and the placebo effect in the three tDCS groups separately. We found a significant correlation in the cathodal tDCS group (*r* = 0.52, *p* = 0.008; significant after Bonferroni correction (*p* = 0.016 (0.05/3)), but not in the anodal and sham tDCS groups (anodal: *r* = 0.33, *p* = 0.11; sham: *r* = 0.45, *p* = 0.027, not significant after Bonferroni correction (*p* = 0.016 (0.05/3))).

**Fig. 4.**
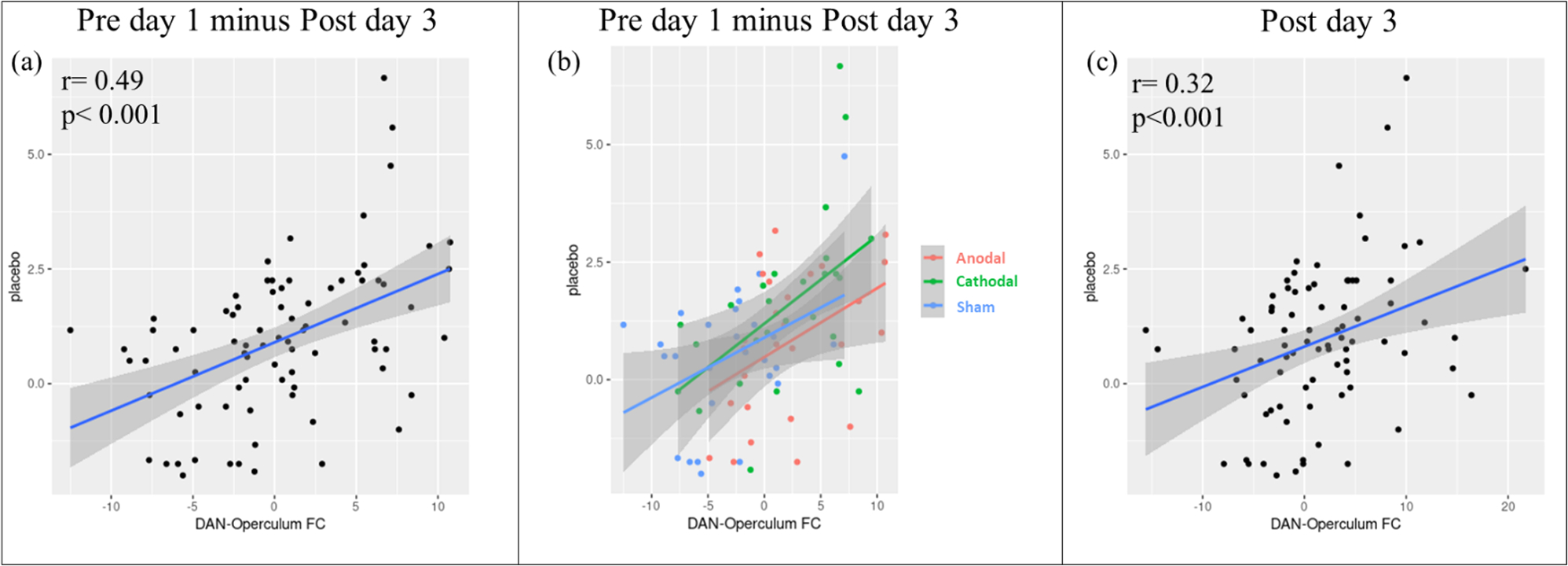
(a) Correlation between the DAN-right parietal operculum FC change and placebo analgesia in the all three tDCS groups in the comparison between post day 3 and pre day 1 (main effect of the stimulation; pre tDCS day 1 minus post tDCS day 3); (b) correlation between the DAN-right parietal operculum FC change (between post day 3 and pre day 1) and the corresponding placebo analgesia in anodal, cathodal, and sham groups separately; (c) correlation between the DAN-right parietal operculum FC change and placebo analgesia in the three tDCS groups in post day 3.

Further, we extracted the average z values of the significant clusters in the post-tDCS resting state after the last tDCS treatment and investigated the association between the FC and the placebo effects. We found a significant positive correlation between the right parietal operculum and DAN (same cluster as above derived from comparison between cathodal and sham groups (*r* = 0.32, *p* < 0.001; significant after Bonferroni correction (*p* = 0.008 (0.05/6)). Correlation plots are reported in Fig. 4.

Finally, a trend was found between the left superior parietal lobule/precuneus and FPN (the significant cluster between the cathodal and sham groups) (*r* = 0.27, *p* = 0.015; not significant after Bonferroni correction (*p* = 0.008 (0.05/6)). No other significant results were found (Fig. 4). More details about the correlation analysis are reported in the Supplementary Materials (Table S3 and S4).

No significant results were found in the correlations between the clusters and the nocebo effect.

## 4. Discussion

In this study, we investigated the modulation effects of different tDCS modes (anodal, cathodal, and sham) on two important brain networks involved in cognitive control, the DAN and FPN, as well as the association between the FC changes evoked by tDCS with the placebo and nocebo effects. We found that compared to sham tDCS, 1) both cathodal and anodal tDCS could increase the FC between the right parietal operculum and DAN, and the cathodal tDCS was also associated with increased FC between the DAN and right postcentral gyrus; 2) anodal tDCS led to increased FC between the FPN and right parietal operculum, while cathodal tDCS was associated with increased FC between the FPN and left superior parietal lobule/precuneus; and 3) the FC increase between the DAN and right parietal operculum (derived from cathodal and sham tDCS comparison) was significantly correlated to placebo analgesic effects.

### 4.1. Dorsal attention network

The DAN is a task-positive network that supports several cognitive functions, such as internal evaluation, working memory, attention, and creativity (Brissenden et al., 2016; Corbetta and Shulman, 2002; Schendan, 2019). It is a bilateral network composed by the intraparietal sulcus (IPS) and the frontal eye fields (FEF) of each hemisphere (Vossel et al., 2014). These areas play a pivotal role in the activation and control of attention (Vossel et al., 2014). Numerous studies (Bushnell et al., 2013; Ossipov et al., 2010) have suggested that attention and distraction can significantly modulate the human pain experience.

We found that anodal and cathodal tDCS led to an increased FC between the DAN and right parietal operculum, cathodal tDCS also showed an increased FC in the DAN-right postcentral gyrus in comparison with the sham group. The correlation analysis showed that the increased FC in the DAN-right operculum (detected in the comparison between cathodal and sham groups) was positively correlated with placebo analgesia in the cathodal tDCS group but not in the anodal and sham tDCS groups.

In addition, the DAN- right parietal operculum FC after tDCS (right before the placebo/nocebo effect testing) was also correlated with placebo analgesia across all subjects. These findings suggest that tDCS, particularly cathodal tDCS, can increase the FC of the DAN that may further modulate placebo effects.

The operculum is the cortical structure adjacent to the insular lobe that plays a role in sensory processing, autonomic and cognitive processing, motor processing, and language. (Mălîia et al., 2018). Like the DAN and FPN, the operculum is also involved in attention and task control (Higo et al., 2011).

Moreover, the parietal operculum houses the secondary somatosensory cortex (S2), while the postcentral gyrus is located in the lateral parietal lobe and constitutes the primary somatosensory cortex (S1). Both S1 and S2 are responsible for the identification and process of different stimuli from the body, including pain (Apkarian et al., 2005; Fomberstein et al., 2013; Johns, 2014). Studies suggest that S1 is the first level of pain perception as it receives the sensory information of pain (Backonja, 1996), whereas S2 receives input from the S1 and preserves and further processes the pain information (Chen et al., 2008; Raju and Tadi, 2021). Studies found that S1 and S2 were involved in pain intensity discrimination (Lockwood et al., 2013). A study using TMS over S1 and S2 showed that S2 was involved in the perception of pain intensity (Lockwood et al., 2013). We found that both S1 and S2 brain regions were activated when we compared high intensity with low intensity heat pain (Kong et al., 2010). Our finding that only the S2 region was correlated with placebo analgesia suggests that S2 may play a more important role over S1 in placebo analgesia. Interestingly, we found that both cathodal and anodal tDCS can increase the placebo analgesic effect, with the cathodal tDCS producing greater placebo analgesia compared with the sham tDCS. Taken together, our findings suggest that tDCS applied at the right DLPFC may work through the DAN – right parietal operculum to enhance the placebo effect.

### 4.2. Frontal parietal network

The FPN is composed of the rostral lateral and dorsolateral prefrontal cortex, and the anterior inferior parietal lobule. Literature suggests that the FPN plays a pivotal role in cognitive/executive control and is actively involved in several cognitive tasks such as working memory, decision making, attention, and reasoning (Chenot et al., 2021; Dixon et al., 2018; Kong et al., 2013; Marek and Dosenbach, 2018). In a previous study (Kong et al., 2013), we found that both the FPN (prefrontal and parietal cortex) and rostral anterior cingulate cortex are involved in the cognitive modulation of pain using a visual cue condition model.

In this study, we found that anodal tDCS increased the FC between the FPN and right parietal operculum, in the region adjacent to (but not overlapping with) the operculum cluster detected in the DAN analysis. As we mentioned above, the S2 is one of the key regions involved in heat pain processing (Apkarian et al., 2005; Fomberstein et al., 2013). Previous studies suggest that the FPN is connected with the DAN for the regulation of the perceptual attention (Dixon et al., 2018). We found that anodal tDCS can increase the FC of the parietal operculum, specifically S2, within both the FPN and DAN, suggesting that tDCS could modulate the connection of S2, which is a key pain processing region, with two interactive cognitive brain networks simultaneously.

It’s worth noting that in this study, the active tDCS produced an effect in the FPN-left supramarginal gyrus, irrespective of its polarity. In fact, we also found that the cathodal mode increased the FC between the FPN and left superior parietal gyrus. The increased FC between the FPN and superior parietal lobule (derived from comparing cathodal and sham tDCS) presented a trend (*p* = 0.015; not significant after Bonferroni correction) in the correlation with placebo analgesia. The superior parietal lobule is involved in multiple brain functions such as attention and working memory (Alahmadi, 2021; Koenigs et al., 2009; Wilkinson, 1992) as well as interpretation of sensory inputs such as touch and pain (Clarke, 1994; Lhomond et al., 2019). The enhanced FC of the FPN and superior parietal gyrus suggests their involvement in tDCS modulation on placebo effects. However, we cannot rule out the possibility that the observed modulation effect in this area may not be solely attributable to the neuromodulatory effect of tDCS stimulation but could also be related to the attentional drive induced by somatosensory stimulation from tDCS.

Overall, our findings suggest that tDCS applied at the right DLPFC triggers FC changes of the DAN and FPN with brain regions involved in pain sensory receiving and processing and stimulus selection and attention. These findings suggest that modulating FC within the DAN and FPN may be a promising approach for enhancing the placebo effect and improving pain management. More research is needed to confirm these findings and to understand the mechanisms by which modulating FC works.

These results are consistent with our previous work (Tu et al., 2021b) which showed that tDCS could boost the placebo effect by altering brain activities during pain.

### 4.3. Limitations

The study has some limitations that should be considered when interpreting the results. The first limitation is that the study was conducted on healthy subjects. It is unclear if the findings can be generalizable to individuals with chronic pain in which the placebo effect may work differently. Also, the brief experimental pain we applied is different from ongoing pain. Further studies that include subjects with specific conditions are needed to test if the effects of modulating FC on the placebo effect would vary depending on the pain condition.

The second limitation is that no MRI data were collected during the tDCS session on day 2. This means that we cannot know if there were any changes in FC within the DAN and FPN on day 2. It is possible that the changes in FC that were observed on day 1 were not sustained.

## 5. Conclusions

We found that tDCS over the rDLPFC could increase the functional connectivity of both the DAN and FPN, which are involved in attention, sensory perception, and information manipulation. The functional connectivity increase between the DAN and right parietal operculum was associated with placebo analgesia in the cathodal tDCS group, suggesting that employing cathodal tDCS might yield outcomes that are more consistent across participants than sham or anodal tDCS. Elucidating how repeated tDCS can modulate the brain network functional connectivity and its association with placebo / nocebo effects may shed light on our understanding of the placebo / nocebo mechanisms, as well as facilitate the development of new strategies for pain management.

## Supplementary Material

1

## Figures and Tables

**Table 1:** Statistical analysis on the DAN and FPN FC differences (pre-tDCS on dayl vs post-tDCS on day 3) across three groups.

Contrast	Brain regions	Cluster size	T peak	MNI coordinates		
				x	y	z

Dorsal Attention Network						

Pre-tDCS day 1 minus Post-tDCS day 3						

Anodal > Sham	Right Parietal Operculum	35	4.32	58	−16	14
Sham > Anodal	No regions survive the threshold					
Cathodal > Sham	Right Postcentral Gyrus	25	4.3	48	− 20	46
	Right Postcentral Gyrus	25	4.03	50	−14	50
	Right Parietal Operculum	24	3.94	54	−16	12
Sham > Cathodal	No regions survive the threshold					

Frontoparietal Network						

Pre-tDCS day 1 minus Post-tDCS day 3						

Anodal > Sham	Right Parietal Operculum	32	3.9	56	− 32	22
Sham > Anodal	No regions survive the threshold					
Cathodal > Sham	Left Superior Parietal Lobule	36	4.33	−12	− 74	40
Sham > Cathodal	No regions survive the threshold					

“Pre day 1″ indicates pre resting state in day 1 before the application of the tDCS; “Post day 3″ indicates the resting state after the tDCS on day 3 (end of the treatment). Results were significant at cluster P_FDR_ ≤ 0.05, corrected at whole brain level. In DAN, cluster from Anodal > Sham contains also right transverse temporal gyrus and right planum temporale. While in FPN, the cluster from Cathodal > Sham includes also the left Precuneus, and the cluster from Anodal > Sham contains also the right planum temporale.

## Data Availability

fMRI data can be requested from the corresponding author. Code for the fMRI preprocessing can be found in https://fmriprep.org/en/stable/index.html. Independent Component Analysis (ICA) code and step can be found on https://web.conn-toolbox.org/fmri-methods/connectivity-measures/networks-voxel-level#h.p_V9IED9jquxDz and in (Calhoun et al., 2001).

## Data Availability

Data will be made available on request.
