# Supplementary material for: Modulation effects of repeated transcranial direct current stimulation on the dorsal attention and frontal parietal networks and its association with placebo and nocebo effects
Source: Neuroimage. Author manuscript; Available in PMC 2024 Jan 5. (PMC10768876; doi:10.1016/j.neuroimage.2023.120433)
Supplement: 1 [file NIHMS1949320-supplement-1.docx]

**Supplementary material**

Modulation effects of repeated transcranial direct current stimulation on the dorsal attention and frontal parietal networks and its association with placebo and nocebo effects

Valeria Sacca^1^, Ya Wen^1^, Sierra Hodges^1^, Jian Kong^1*^

^1^ Department of Psychiatry, Massachusetts General Hospital and Harvard Medical School, Charlestown, MA 02129, USA

*Demographics and behavior results*

All 81 participants who completed the study were included in the analysis. No differences were found in age (p= 0.84) or gender (p= 0.82; see Table S1). The participants were then divided into three groups based on the tDCS mode (anodal, cathodal, and sham). We used an expectancy manipulation model for inducing expectations of three inert creams labeled as “lidocaine” (inducing expectations for decreased pain), “capsaicin” (inducing expectations for increased pain), and “neutral” (for control). Conditioning was obtained by decreasing, increasing, or using a moderate noxious stimulus intensity on the subject’s forearm.

Placebo and nocebo responses were analyzed using analysis of covariance (ANCOVA) with age, cream randomization, and difference in expectations for relief scale (ERS; extracted in Session 2 before and after the expectancy manipulation) as covariates. ANCOVA was performed separately for placebo and nocebo responses with the tDCS group as the fixed factor. In addition, State-Trait Anxiety Inventory (STAI) scores were included as a covariate for the analysis of the nocebo response. Post hoc analyses were performed using Tukey test. Statistical analyses were performed using R.

The data analysis showed that participants reported significantly different pain perceptions (rated with the Gracely scale) across the three heat pain intensities (p<0.001). However, this difference was not found across the tDCS groups (p= 0.44). Moreover, participants’ expectations of the analgesic effect of the lidocaine cream and of the hyperalgesic effect of the capsaicin cream (rated with a scale from 0 to 10) were significantly increased in all the groups after the expectancy manipulation. Based on these results, we assumed that all the participants within the three groups had similar levels of conditioning during the experiment.

Considering placebo and nocebo effects, participants in the two active tDCS groups showed significant placebo analgesia (p<0.05 for anodal and p<0.001 for cathodal, paired-sample t test) but not significant nocebo hyperalgesia (p= 0.22 for anodal and p= 0.07 for cathodal, paired-sample t test). Participants from the sham group also reported significant placebo and nocebo effects, demonstrating the validity of the experimental design for inducing those effects.

In addition, the comparison between the three groups had significant main effects of the active tDCS groups on placebo analgesia (i.e., lidocaine versus neutral; p= 0.034) and nocebo hyperalgesia (i.e., capsaicin versus neutral; p= 0.044). Post-hoc comparisons revealed that cathodal tDCS significantly increased placebo analgesia in comparison to the sham group (p_Tukey_= 0.028), and that anodal tDCS significantly inhibited nocebo hyperalgesia compared to the sham group (p_Tukey_= 0.041).

*Quality control*

Figure S1 summarizes the tSNR in pre tDCS day 1 and post tDCS day 3 rs-fMRI sequences among the three groups. ANOVA was performed to assess any statistical differences. In both time points, tSNR did not differ across the three tDCS groups (p= 0.8 in pre tDCS day 1 and p= 0.37 in post tDCS day 3). In addition, the tSNR did not differ across the two rs-fMRI sequences (paired t-test, p= 0.3) when considering all the subjects.


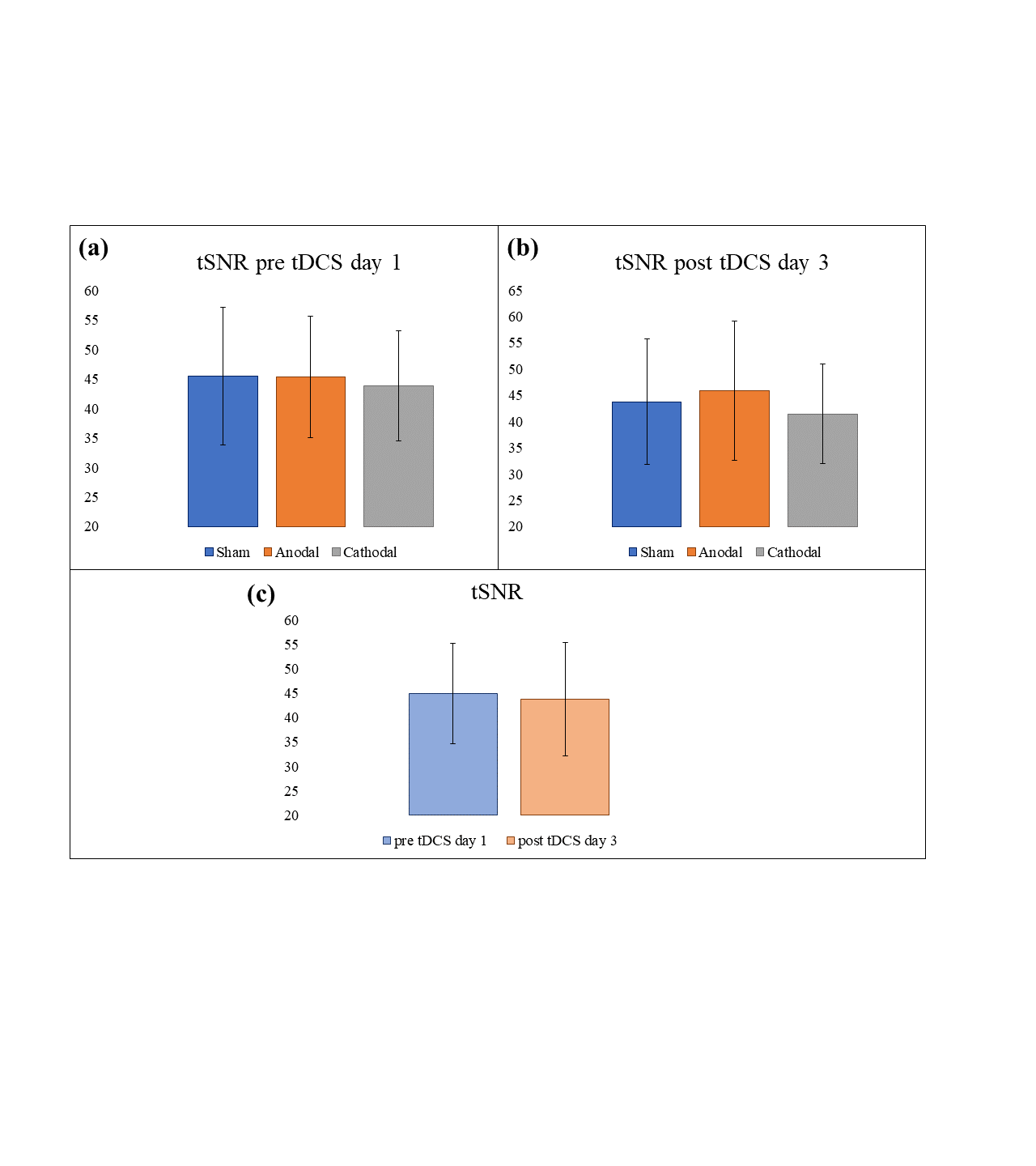


Figure S1: The mean and standard deviation of tSNR extracted in pre tDCS day 1 (a) and post tDCS day 3 (b) for each group; (c) mean and standard deviation of the two time points considering all the subjects.

Table S1: demographic parameters among the three groups.

|  | Anodal tDCS | Cathodal tDCS | Sham tDCS |
| --- | --- | --- | --- |
| Number | 27 | 27 | 27 |
| Age | 27.4 ± 6.3 | 26.9 ± 5.9 | 27.9 ± 7.1 |
| Gender (F/M) | 14/13 | 16/11 | 14/13 |

Table S2: behavioral results in Session 2 and Session 5

|  | Anodal tDCS | Cathodal tDCS | Sham tDCS |
| --- | --- | --- | --- |
| Session 2 | | | |
| Pain ratings for low painful stimuli | 6.05 ± 2.67 | 4.54 ± 2.04 | 5.08 ± 2.14 |
| Pain ratings for moderate painful stimuli | 9.31 ± 1.97 | 8.84 ± 2.54 | 8.97 ± 2.34 |
| Pain ratings for high painful stimuli | 14.52 ± 2.67 | 14.56 ± 2.33 | 14.52 ± 2.20 |
| Changes of expectation for pain decrease | 1.70 ± 2.89** | 2.30 ± 2.45*** | 1.67 ± 2.59** |
| Changes of expectation for pain increase | 1.78 ± 2.22*** | 2.00 ± 2.47*** | 2.48 ± 2.41*** |
| Session 5 | | | |
| Placebo analgesia | 0.81 ± 1.54* | 1.55 ± 1.81*** | 0.50 ± 1.47* |
| Nocebo hyperalgesia | 0.44 ± 1.82 | 0.84 ± 2.32 | 1.13 ± 1.46*** |

Values are reported in mean ± standard deviation. *p* values are corrected for multiple comparisons using False Discovery Rate. **p* < 0.05; ** *p* < 0.01; *** *p* < 0.001, two-tail t test; participants in the sham tDCS group had significant placebo analgesia (p < 0.05 for one-tail t test).

*Correlation analysis*

The slopes for each of the three correlations were extracted and analyzed. Results are reported in Table S3. The t-value is the measure of how many standard deviations the slope coefficient estimate is from 0, while the p-value represents the probability of observing any value equal or larger than t. Considering Bonferroni correction applied to the three different correlations, the slope coefficient is statistically significant only considering the cathodal group (p= 0.006, significant after Bonferroni correction (p=0.05/3= 0.016)).

Table S3: correlation outputs.

| group | slope | standard error | sample size | t value | p-value |
| --- | --- | --- | --- | --- | --- |
| sham | 1.2 | 0.57 | 27 | 2.15 | 0.041 |
| cathodal | 1.4 | 0.47 | 27 | 2.99 | 0.006 |
| anodal | 1.2 | 0.52 | 27 | 2.31 | 0.029 |

Furthermore, we performed the two sample t tests between the slopes of the three groups as suggested by the reviewer. No significant results were found (Table S4). However, cathodal tDCS reported a non-significant steeper slope in comparison to the sham and anodal tDCS (as shown in Figure 4b and Table S4).

Table S4: two sample t-tests performed on the slopes between the three groups.

| groups | p value |
| --- | --- |
| cathodal vs sham | 0.78 |
| cathodal vs anodal | 0.77 |
| anodal vs sham | 1 |
